# Abnormal visual gain control and excitotoxicity in early-onset Parkinson’s disease *Drosophila* models

**DOI:** 10.1152/jn.00681.2017

**Published:** 2017-11-15

**Authors:** Marc M. Himmelberg, Ryan J. H. West, Christopher J. H. Elliott, Alex R. Wade

**Affiliations:** ^1^Department of Psychology, The University of York, York, United Kingdom; ^2^Department of Biology, The University of York, York, United Kingdom

**Keywords:** *Drosophila*, excitotoxicity, gain control, linear discriminant analysis, Parkinson’s disease, SSVEPs

## Abstract

The excitotoxic theory of Parkinson’s disease (PD) hypothesizes that a pathophysiological degeneration of dopaminergic neurons stems from neural hyperactivity at early stages of disease, leading to mitochondrial stress and cell death. Recent research has harnessed the visual system of *Drosophila* PD models to probe this hypothesis. Here, we investigate whether abnormal visual sensitivity and excitotoxicity occur in early-onset PD (EOPD) *Drosophila* models *DJ-1α*^Δ^*^72^, DJ-1β*^Δ*93*^, and *PINK1^5^*. We used an electroretinogram to record steady-state visually evoked potentials driven by temporal contrast stimuli. At 1 day of age, all EOPD mutants had a twofold increase in response amplitudes compared with *w̄* controls. Furthermore, we found that excitotoxicity occurs in older EOPD models after increased neural activity is triggered by visual stimulation. In an additional analysis, we used a linear discriminant analysis to test whether there were subtle variations in neural gain control that could be used to classify *Drosophila* into their correct age and genotype. The discriminant analysis was highly accurate, classifying *Drosophila* into their correct genotypic class at all age groups at 50–70% accuracy (20% chance baseline). Differences in cellular processes link to subtle alterations in neural network operation in young flies, all of which lead to the same pathogenic outcome. Our data are the first to quantify abnormal gain control and excitotoxicity in EOPD *Drosophila* mutants. We conclude that EOPD mutations may be linked to more sensitive neuronal signaling in prodromal animals that may cause the expression of PD symptomologies later in life.

**NEW & NOTEWORTHY** Steady-state visually evoked potential response amplitudes to multivariate temporal contrast stimuli were recorded in early-onset PD *Drosophila* models. Our data indicate that abnormal gain control and a subsequent visual loss occur in these PD mutants, supporting a broader excitotoxicity hypothesis in genetic PD. Furthermore, linear discriminant analysis could accurately classify *Drosophila* into their correct genotype at different ages throughout their lifespan. Our results suggest increased neural signaling in prodromal PD patients.

## INTRODUCTION

Parkinson’s disease (PD) is the second most common progressive neurodegenerative disorder, affecting ~0.2–3% of the population, with an increased prevalence in those aged over 50 (Clarke 2007; de Rijk et al. 1997). PD is thought to stem from the pathophysiological degeneration and subsequent loss of dopaminergic neurons within the pars compacta of the substantia nigra, a basal ganglia structure that plays a key role in movement (Clarke 2007). It is hypothesized that neuronal death in PD is caused by an excitotoxic mechanism, in which neuronal hyperactivity leads to neurodegeneration. Neuronal hyperactivity causes an increase in demand for ATP from mitochondria, leading to oxidative stress and eventual neuronal death (Beal et al. 1993; Surmeier et al. 2017). In both mammals and invertebrates, neuronal responses are regulated by a tightly linked network of excitatory and inhibitory gain control mechanisms that, collectively, we refer to as “normalization” (Carandini and Heeger 1994, 2011; Carandini et al. 1997; Single et al. 1997). Normalization mechanisms can be measured across the animal kingdom by a range of methods, including steady-state visually evoked potential (SSVEP) recordings, a sensitive technique commonly used to measure the amplitude of neural population responses to periodic flickering stimuli (Busse et al. 2009; Norcia et al. 2015; Regan 1966; Tyler et al. 1978).

In *Drosophila*, SSVEP recordings are collected from the surface of the eye and can be made in both healthy and PD mutant strains (Afsari et al. 2014; West et al. 2015a, 2015b). Previously we have shown that young flies carrying the late-onset gain-of-function PD mutation *LRRK2-G2019S* showed increased visual contrast sensitivity to full-field flicker stimuli, reflecting a failure in regulation of neural activity (i.e., abnormal gain control or normalization) at 1 day of age (Afsari et al. 2014). This regulatory failure is followed by a decline in visual function over time, with physiological and anatomical degeneration in older *LRRK2-G2019S Drosophila* (Hindle et al. 2013; Mortiboys et al. 2015).

Feeding *LRRK2-G2019S Drosophila* with BMPPB-32, a kinase inhibitor specifically targeted at *LRRK2*, restored normal contrast sensitivity at both 1 and 14 days of age, indicating that both the early neuronal hypersensitivity and the subsequent neurodegeneration are due to abnormal kinase domain activity (Afsari et al. 2014). Vision loss was accelerated by increasing neural activity via photic stimulation of the *Drosophila* visual system using flashing LED lights. Together, these findings support an excitotoxicity theory of the *LRRK2-G2019S* form of PD. This excitotoxicity theory of PD has also found support in rodent models of the *G2019S* mutation (Longo et al. 2014; Matikainen-Ankney et al. 2016; Ponzo et al. 2017; Sloan et al. 2016; Volta et al. 2017).

We have previously demonstrated that linear discriminant analysis (LDA) is a useful tool in the analysis of SSVEP data obtained from *Drosophila* (West et al. 2015a). Here, our findings indicated differences in SSVEP amplitude both between and within wild-type flies and in early-onset PD (EOPD) mutants, in response to spatiotemporal patterns. These differences had enough statistical regularity for LDA to accurately discriminate between genotypes. Compared with wild-type controls, qualitative observations indicated an elevation in SSVEP response in 1-day-old EOPD flies. Although LDA has diagnostic utility, it does not allow for the quantification of directional differences in such responses. Having established this method, we now seek to expand on this and investigate abnormal gain control and excitotoxicity in EOPD models.

Is excitotoxicity a general feature of all *Drosophila* PD mutants? If so, it would suggest that, rather than being an epiphenomenon of some metabolic dysfunction that causes PD, the excitotoxicity itself is central to the disease. In the present study, we use SSVEP techniques combined with principal components analysis (PCA), general linear modeling, and multivariate classification analysis to investigate abnormal gain control and excitotoxicity in EOPD *Drosophila* models. We hypothesized that abnormal gain control would occur in young *Drosophila* carrying EOPD mutations due to disease-related changes in retinal dopaminergic neurons, reflected by increased SSVEP amplitudes in 1-day-old EOPD *Drosophila* mutants. We also hypothesized that abnormal gain control would cause an excitotoxic cascade in older EOPD *Drosophila*. Consequently, we expected to observe a decrease in SSVEP amplitudes at later ages. Finally, we wondered whether all mutations affected neuronal gain control in the same manner or whether there were subtle mechanistic variations that could be used to differentiate the genotypes. To address this, we used LDA based on SSVEP responses to a range of temporal modulation rates and contrast levels to attempt to classify flies into their correct genotypic class at different points throughout their lifespan. The greater the differences in the gain control profiles across genotypes, the greater the accuracy we expected from this classification.

We found that SSVEP response amplitudes to spatial stimuli are significantly increased in EOPD mutants at 1 day of age, indicating that neuronal gain control is abnormal in these animals. Generating additional neuronal stress by exposing flies to randomly pulsating light for 7 days resulted in a profound loss of vision in all PD mutants, supporting the excitotoxicity model of PD. Finally, there are robust differences between the temporal contrast response profiles of the different PD mutants that allow our multivariate classification algorithms to classify flies into their respective genotypes at well above chance levels throughout their lifespan.

## MATERIALS AND METHODS

### Drosophila Stocks and Maintenance

*Drosophila* were raised in a 12:12-h light-dark (LD) cycle at 25°C on standard food consisting of agar (1% wt/vol), cornmeal (3.9%), yeast (3.7%), and sucrose (9.4%). All flies were outcrossed and stabilized where appropriate to remove any naturally occurring mutations. Three EOPD mutations (*DJ-1α*^Δ^*^72^, DJ-1β*^Δ^*^93^*, and *PINK1^5^*), one knockout of the fly *LRRK2* homologue (*dLRRK^ex1^*), and one wild-type control genotype (*w^1118^*, herein *w̄*) were deployed. The *w̄* strains were gifted by Sean Sweeney. *PINK1^5^* and *dLRRK^ex1^* strains were obtained from the Bloomington *Drosophila* Stock Centre (Bloomington, IN), and *DJ-1α*^Δ^*^72^* and *DJ-1β*^Δ^*^93^* strains were kind gifts from Alex Whitworth. Male flies all had white eyes and were tested at 1, 7, 14, 21, and 28 days posteclosion.

### Preparation of Drosophila for Testing

Male flies were collected within 8 h of eclosion and transferred to a new vial of standard food that additionally contained nipagin (0.1% wt/vol). Flies were maintained in these vials and transferred to fresh food weekly. Flies were kept in a 12:12-h LD cycle at 25°C until they had reached appropriate age for testing.

### Photic Stress

To explore as to whether an increase in neural demand resulted in a decrease in SSVEP amplitudes, all *Drosophila* genotypes were exposed to a photic stressor condition (Afsari et al. 2014; Hindle et al. 2013). Male flies were collected within 8 h of eclosion and transferred to a new vial of standard food containing nipagin. These flies were maintained within a 29°C incubator with irregularly pulsating LED lights at ~1.5-s intervals to force the *Drosophila* visual system to adapt to new light levels and increase photoreceptor response. Flies were maintained here for 7 days, as this was the age at which *G2019S* mutants had previously shown visual loss (Hindle et al. 2013). Ten flies of each genotype were tested (except for *DJ-1α*^Δ^*^72^* where eight were tested) (*N* = 48).

### Preparation for Electroretinogram

On the day of testing, flies were collected using a pooter and aspirated into a shortened pipette. Once the fly’s head was protruding from the tip of the pipette, it was restrained by placing a small layer of nail varnish on the back of the fly’s neck. Two pipettes at a time were mounted onto a customized *Drosophila* electroretinogram (ERG) recording system, with both flies placed 22 cm away from the dual display monitors (West et al. 2015a). ERG recordings were made through hollow drawn-glass electrodes containing simple saline (130 mM, NcCl, 4.7 mM KCl, 1.9 mM CaCl_2_) connected to a high-impedance amplifier [LF356 op-amp in the circuit (Fig. 7 of Ogden 1994)] via thin silver wires. The reference electrode was inserted gently onto the *Drosophila* proboscis, and the recording electrode was placed on the surface of the right eye. Ten unique flies of each genotype at each age were tested (total *N* = 250).

### Stimuli

Stimuli were contrast-reversing achromatic sine wave gratings with a range of Michelson contrasts (Michelson 1927) and temporal frequencies. Spatial frequency was held at 0.056 cycles per degree as this had previously been found to be the optimal spatial frequency to measure SSVEP recordings from *Drosophila* (West et al. 2015a). Stimuli were generated using the Psychophysics Toolbox on a Windows 7 PC and were displayed on dual 144-Hz LCD monitors (XL240T, BenQ, Tiwam). Stimuli swept through unique combinations of eight levels of temporal frequency (1, 2, 4, 6, 8, 12, 18, and 36 Hz) and eight levels of contrast (1, 4, 8, 16, 32, 64, 99%) to generate 64 different combinations of temporal contrast stimuli. Parameter combinations were presented in a random order for an 11 s trial, with a 4 s inter-stimulus interval. The first second of each trial was removed before analysis to remove onset transients. Each parameter combination was presented three times per fly to create a ~1-h recording session.

### Analysis

#### Steady-state visually evoked potentials.

**Fig. 1.**
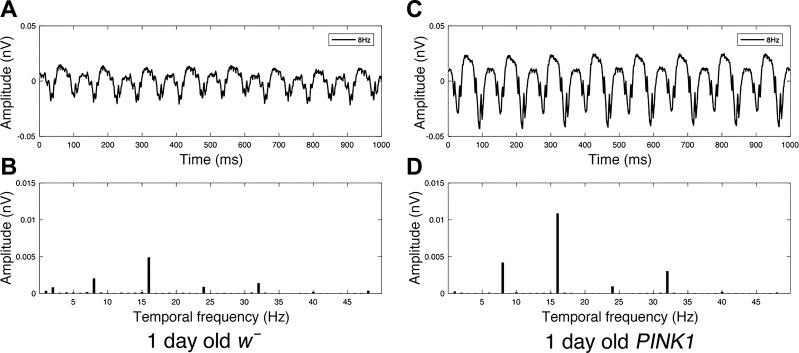
Time-domain steady-state visually evoked potential (SSVEP) with a stimulus input frequency of 8 Hz contains 16 “reversals” per second and can be decomposed into a SSVEP response spectrum with peaks at multiples of the input frequency. In *A*, we present an averaged time-domain SSVEP response from a *w̄* fly to 99% contrast reversing sine grating over 1,000 ms, modulating at 8 Hz, whereas *B* shows Fourier amplitudes decomposed from Fourier transform the 8-Hz waveform in *A*, with peaks occurring at multiples of our input frequency (8 Hz, 16 Hz, 24 Hz, 32 Hz, 40 Hz). The same is shown in *C* and *D* for a *PINK1^5^* PD-mutant fly.

The periodic modulation of a contrast reversing grating evokes SSVEPs with a phase-locked, periodic time course which is analyzed most conveniently in the frequency domain (see Fig. 1, *A* and *C* for examples of SSVEP response from *w̄* and *PINK1^5^* mutants). For a single, contrast-reversing grating, the ERG records responses from both the photoreceptors and the subsequent neuronal signaling pathways (Afsari et al. 2014). Individual photoreceptors track the luminance modulations of the grating bars at the input frequency (*F*1), but, because the signal elicited by a grating is a population average of photoreceptors driven by different transition polarities (some dark→light, some light→dark), the overall photoreceptor contribution is largely self-cancelling. Residual responses at *F*1 arise from asymmetries in photoreceptor sampling of the relatively low spatial frequency grating. The majority of the signal is composed of the transient responses arising from the visual neurons which are confined to even multiples of the input frequency. Of these responses, the second harmonic is by far the largest and we restrict our analyses to 2*f* for each input frequency. A coherently averaged (phase-sensitive) Fourier amplitude was calculated for each temporal frequency and contrast combination by averaging complex frequency-domain data obtained for each condition over three runs (see Fig. 1, *B* and *D* for examples of Fourier amplitudes from *w̄* and *PINK1^5^* mutants). Due to the phase-locked nature of VEPs, coherent averaging preserves the signal while phase-randomized noise sums to zero (Norcia et al. 2015). This results in a high signal-to-noise ratio for SSVEP recordings.

#### Linear discriminant analysis.

**Fig. 2.**
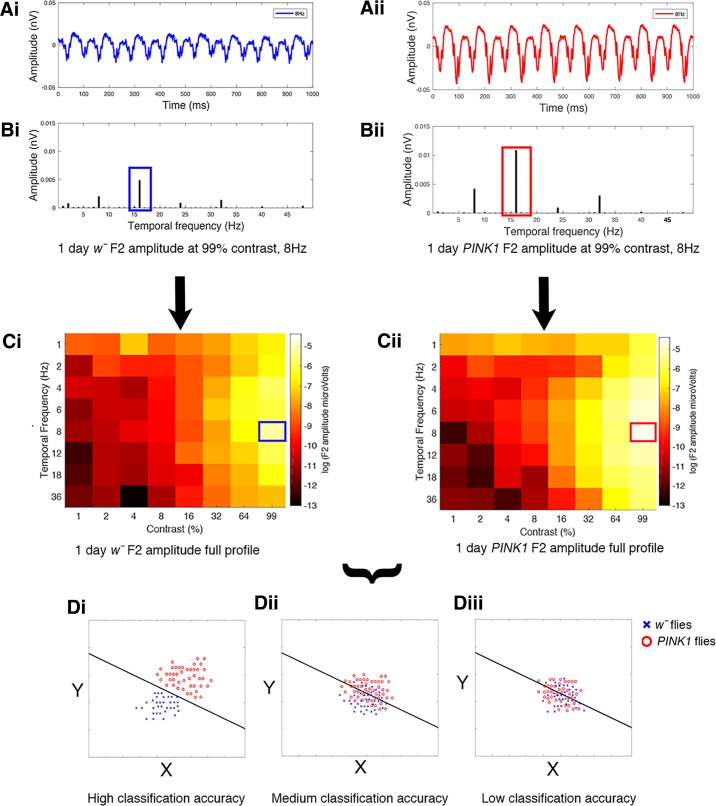
Analysis path for linear discriminant analysis (LDA). The raw ERG (electroretinogram) response to 64 different stimuli is collected, here from a control (wild-type) *w̄* fly and an early-onset Parkinson’s disease (EOPD) (*PINK1^5^)* fly (*A*). For each stimulus, Fourier analysis is used to measure the response of the fly at the second harmonic (2*f*) (*B*). Each fly is exposed to 64 stimuli, each with a known contrast and temporal frequency. The heat map (*C*) represents the amplitude of the second harmonic at each stimulus condition. In this simple case, with just 2 genotypes at 1 time point, the LDA is applied to the data from both genotypes and determines the equation that best separates the data into 2 classes based on the 64 responses. Three outcomes could be envisaged — an optimal separation of the data. *Di*: a clear line separates the data, or a partial separation (*Dii*), or no difference (*Diii*), all the data are mixed). In this portrayal, the graph plots *X* and *Y* which will be calculated from the 64 Fourier results by the LDA algorithm. In the more complex data set explored below, 5 genotypes and 5 ages were sampled, leading to a multidimensional “cloud” of data which can still be separated by a (more complex) set of linear equations.

We assessed LDA as a tool to accurately assign flies into their correct genotype based on multivariate visual response profiles. We used ERG measurements recorded in response to 64 combinations of contrast and temporal frequency, thus providing a 64-dimensional data set to input into the LDA. Each fly was therefore located in a 64-dimensional space. Flies that showed similar responses to these combinations of contrast and temporal frequency clustered together in this space. Thus, if different classes showed different visual responses, unique clusters for each class would form in this 64-dimensional space. The LDA algorithm then attempted to identify a single linear boundary between these clusters and classified each fly into a genotypic class by asking which side of this linear boundary the fly was situated. The accuracy of the LDA algorithm depends on the degree of separation between the genotypic clusters in the multidimensional feature space. This is further expanded on in Fig. 2, where we illustrate the process of raw data collection through to a range of possible classifications.

## RESULTS

### Early-Onset PD Temporal Contrast Profile Amplitudes Are Larger Than Controls

**Fig. 3.**
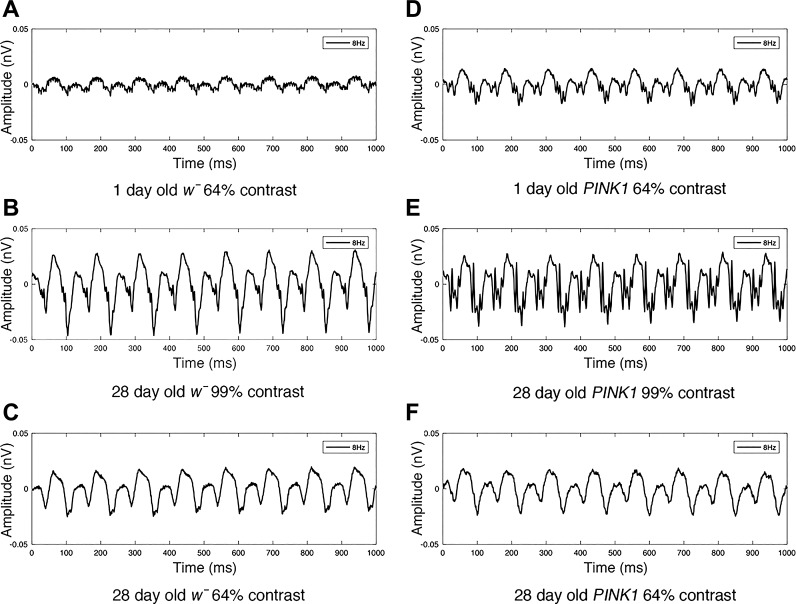
We use the ERG to obtain accurate SSVEP measurements from both wild-type and PD *Drosophila* mutants at different contrasts and ages. In *A*–*F* we present exemplar ERG responses at 8 Hz obtained from *w̄* and *PINK1^5^* PD mutants at 1 and 28 days of age, and at 64 and 99% contrast. SSVEP waveform peak amplitude increases with increasing contrast.

**Fig. 4.**
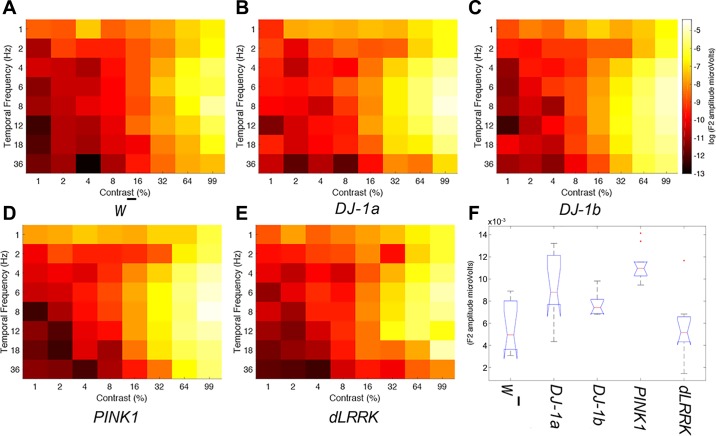
EOPD mutants show steeper response amplitudes at 1 day of age. *A*–*E*: mean response amplitudes from all *Drosophila* genotypes (*n* = 10 for each genotype). *Drosophila* exhibit visual tuning to temporal frequency and contrast, with peak sensitivity at 6–8 Hz temporal frequency and 99% contrast. Furthermore, the maps appear to show subtle differences outside of peak regions between 12 and 36 Hz at 1–8% contrast. Profiles indicate that EOPD mutants have larger response amplitudes at “peak sensitivity” regions. *F*: boxplot of the 2*f* peak response at 99% contrast and 8 Hz for each genotype.

A series of exemplar raw SSVEP responses from both *w̄* and *PINK1^5^* mutants at different ages and stimulus contrasts is illustrated in Fig. 3. Average Fourier amplitudes at 2*f* for each temporal contrast combination for each genotype are illustrated in Fig. 4. Higher peak response amplitudes are represented by lighter colors and lower amplitudes by darker colors. Visual response changes as a function of both contrast and temporal frequency, with responses in both wild-type and EOPD models peaking at high contrast (99%) and an intermediate temporal frequency (6–8 Hz).

### Principal Components Analysis

**Fig. 5.**
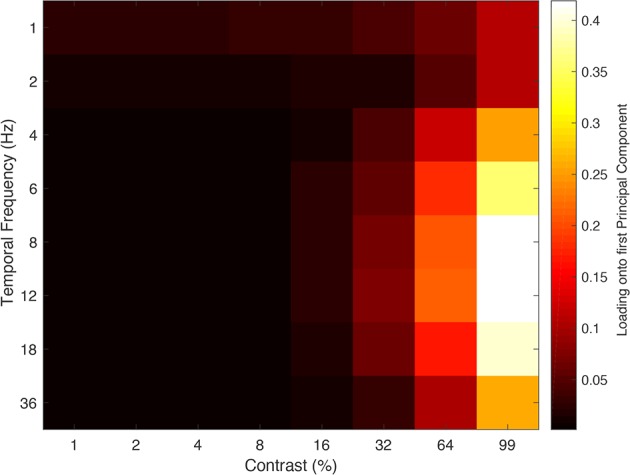
High contrast (99%) and intermediate temporal frequency combinations (6–18 Hz) conditions exhibit the strongest loading onto the first principal component (PC). The entire data set (*N* = 250) is run through the PCA simultaneously to ensure that it is scaled by the same eigenvalue. Brighter colors represented a higher loading onto the first PC and darker colors a lower loading.

We computed a PCA on the full data set (*N* = 250) (See Fig. 5). This allowed us to retain just those principal components (PCs) that explain significant amounts of the overall variance, simplifying our 64-dimensional data significantly (Jolliffe and Cadima 2016; West et al. 2015a). Our first PC explained 89.9% of total variance within the data set and the univariate analysis that follows is based on the amplitude of this component, while the multivariate analysis later in the paper is performed on the full data set.

### Main Effects

A 5 × 5 between-groups ANOVA was performed on the first PC score (representing SSVEP amplitude) to assess whether there was a difference in SSVEP amplitudes between *Drosophila* genotypes or ages. The analysis found a significant main effect of genotype, *F*(4,225) = 21.428, *P* < 0.001, indicating a difference in response amplitude between the five genotypes, when collapsed over age. The analysis also found a significant main effect of age *F*(4,225) = 5,558, *P* < 0.001, indicating a difference in response amplitude between the five ages, when collapsed over genotype. Finally, there was a significant interaction effect *F*(16,225) = 2.984, *P* < 0.001, indicating that response amplitude differed between genotype depending on age. A simple effects analysis was performed to tease out differences in our conditions and explore our interaction effect.

### Simple Effects Analysis Comparing Between Genotypes Within Each Age Group

**Fig. 6.**
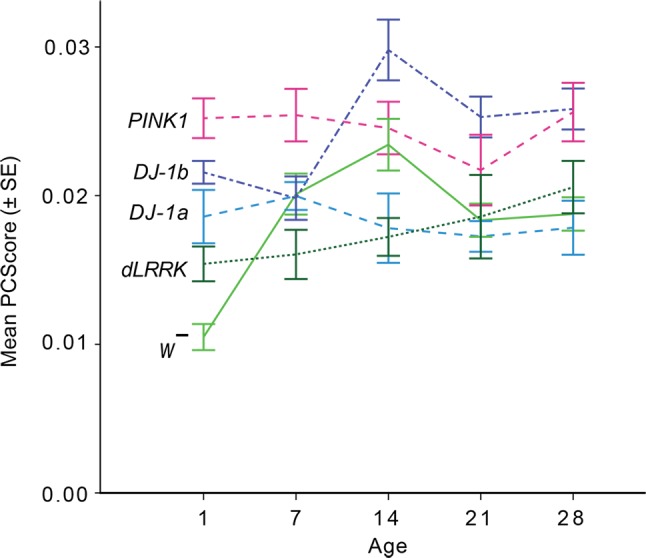
One-day-old EOPD flies show increased SSVEP response amplitudes compared with control flies (*w̄*). Mean PC score (representing response amplitude) as a function of age for 5 *Drosophila* genotypes (*n* = 10 for each genotype/age group). Error bars show ± 1SE.

A simple effects analysis was undertaken to explore differences in the SSVEP amplitudes of *Drosophila* genotypes within each age group, with Sidak corrections applied to all possible comparisons. The SSVEP amplitudes of each genotype as a function of age are illustrated in Fig. 6, and all corresponding *P* values are presented in appendix Table A1. Analysis revealed that at 1 day of age, all EOPD mutations (i.e., excluding *dLRRK^ex1^*) had significantly higher SSVEP amplitudes compared with *w̄* control flies (*P* < 0.01). When comparing between 1-day-old PD mutants, *PINK1^5^* produced significantly higher SSVEP amplitudes compared with both *DJ-1α*^Δ^*^72^* (*P* < 0.05) and *dLRRK^ex1^* mutants (*P* < 0.01). There were no other significant differences in the SSVEP amplitudes of PD mutants. The larger amplitudes of EOPD mutants did not hold over later ages as wild-type response increased at 7 days of age (see Fig. 6). However, differences between the SSVEP amplitudes of PD mutants was found at these later ages. At 7 days of age *PINK1^5^* mutants produced significantly higher amplitudes compared with *dLRRK^ex1^* (*P* < 0.005), whereas at 14 days of age *DJ-1β*^Δ^*^93^* had significantly higher amplitudes compared with *DJ-1α*^Δ^*^72^* (*P* < 0.001*)* and *dLRRK^ex1^* (*P* < 0.001) mutants. This trend continued at 21 days of age, with *DJ-1β*^Δ^*^93^* continuing to show higher SSVEP amplitudes compared with *DJ-1α*^Δ^*^72^* (*P* < 0.01) and *dLRRK^ex1^* (*P* < 0.05). At 28 days of age, *DJ-1β^Δ93^* (*P* < 0.01) and *PINK1^5^* (*P* = 0.01) produced significantly higher SSVEP amplitudes compared with *DJ-1α^Δ72^*.

### Simple Effects Analysis Comparing Between Age Group Within Each Genotype

A simple main effects analysis was undertaken to explore differences in the SSVEP amplitudes within each *Drosophila* genotype over its lifespan, with Sidak corrections applied to all possible comparisons. The *P* values for all simple effects are presented in appendix Table A2. Analysis revealed that *w̄* response amplitudes increased between 1 and 7 days of age (*P* = 0.001); however there was no significant difference when comparing between further consecutive ages within this genotype; thus visual response held stable between 7 to 28 days of age. There was a significant increase in *DJ-1β*^Δ^*^93^* response amplitudes between 7 and 14 days of age (*P* < 0.001), which then held steady from 14 to 28 days of age. There was no significant difference in response amplitudes within *DJ-1α*^Δ^*^72^*, *PINK1^5^* or *dLRRK^ex1^* at any consecutive ages between 1 and 28 days.

### Increased Demand for Energy in the Visual System Leads to Loss of Visual Response in Old PD Flies

While we demonstrated that abnormal gain control occurs in 1-day-old EOPD mutants, at later ages, responses were comparable to those of wild-type flies (*w̄*). This represents a difference between EOPD mutant flies and flies mimicking the late-onset *LRRK2-G2019S* mutation, where responses fall to zero at later ages (Hindle et al. 2013). We hypothesized that maintaining our *Drosophila* stocks at 25°C and a 12:12-h LD cycle did not produce enough neuronal demand on the visual system to see any effect. To test this hypothesis, we increase the demand for energy by exposing *Drosophila* to irregular ~1.5 s flashes of light of at random periodic intervals over 7 days. Here, we hypothesize that the abnormal gain we have observed in young EOPD flies will interact with a visually induced increase in neural demand to cause an excitotoxic cascade.

**Fig. 7.**
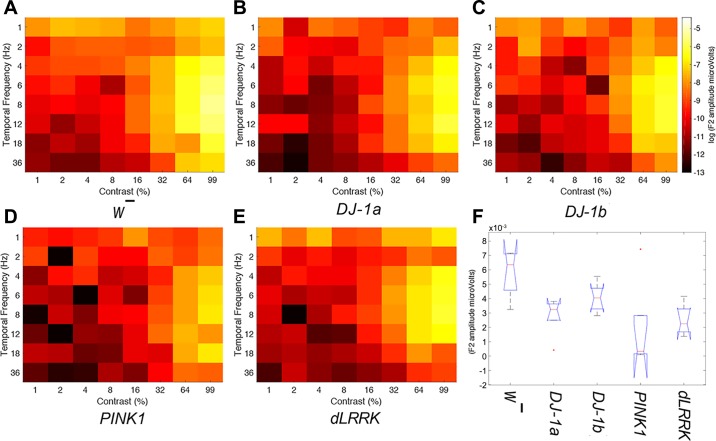
All EOPD mutants show perturbations in response amplitudes after exposure to pulsating light, indicating a decrease in temporal contrast sensitivity (*n* = 10 per genotype). *A*–*E*: mean response amplitudes from all *Drosophila* genotypes after 7 days of visual stimulation (each genotype *n* = 10, except *DJ-1α*^Δ^*^72^* *n* = 8). Same scale as Fig. 3. *F*: boxplot of the 2*f* peak response at 99% contrast and 8 Hz.

Observation of temporal contrast response profiles (see Fig. 7) indicated a profound reduction in SSVEP amplitudes across temporal frequency and contrast combinations for PD mutants (but not wild-type flies) after 7 days exposure to photic stress.

**Fig. 8.**
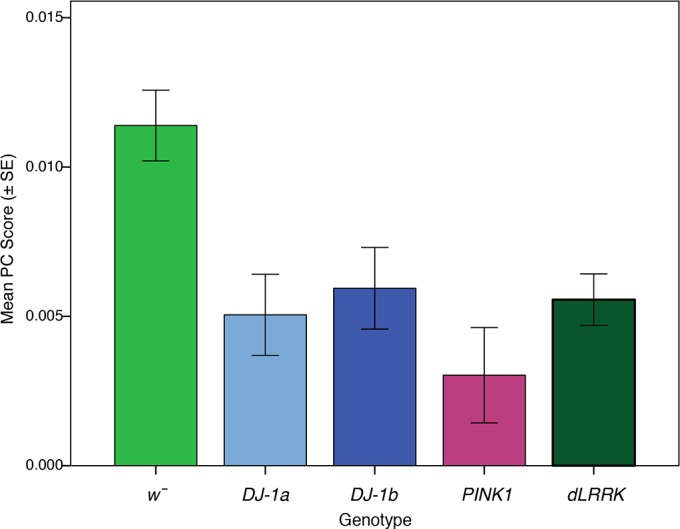
Visual loss occurs in all Parkinson’s disease (PD) mutants after 7 days of exposure to pulsating light. Mean PC Score of 5 *Drosophila* genotypes after 7 days exposure (each genotype *n* = 10, except *DJ-1α*^Δ^*^72^* *n* = 8).

A one-way between-groups ANOVA was performed on the first PC score (representing SSVEP amplitude) extracted via the PCA analysis to assess whether there was a significant difference in visual response between five *Drosophila* genotypes after they had been exposed to 7 days of photic stress. The analysis found a significant main effect of genotype, *F*(1,43) = 5.965, *P* = 0.001, η^2^ = 0.357, indicating a difference in response amplitude between the five genotypes. Pairwise comparisons revealed that all PD mutants produced significantly lower SSVEP amplitudes compared with *w̄* control flies (*P* < 0.05), indicating an interaction between visual stimulation and *Drosophila* genotype on visual response amplitudes (see Fig. 8). There was no significant difference between the PD mutants’ SSVEP responses.

### Linear Discriminant Analysis Classifies Flies into Their Correct Genotypic Class

Thus all EOPD mutants show both an early increased visual response and a loss of vision after 7 days of visual stimulation, compared with *w̄* control flies.

In the presentation of our data so far, we utilized PCA to reduce the dimensionality in our data to a single variable, thereby removing any nuanced differences between full *Drosophila* temporal contrast profiles. We now explore how LDA can use the additional small but significant sources of variation in our SSVEP data to classify *Drosophila* into their correct genotypic class and age group.

LDA is a statistical method that aims to answer both binary and multiclass classification problems by seeking linear combinations of variables that best explain the variance within the data, working under the assumption that unique classes generate unique Gaussian distributions (Izenman 2008). We assess the accuracy of our LDA in two ways. First, we use a standard linear classifier (Fisher 1936) as implemented in MATLAB’s (MathWorks, MA; 2017) “classify” function to conduct a leave-one-out (LOO) analysis, where the classifier receives training data from all flies to be assessed except one, then we measure the classifiers accuracy in classifying the excluded fly. This fly is resubstituted and the classification is repeated for every fly in the data set to return a generalized LOO accuracy. Second, we use MATLAB’s classification function “fitcdiscr” to fit an LDA model to our raw 64-dimensional data. We then use Monte Carlo resampling methods to produce three estimates of accuracy: an overall model accuracy, an N-way classification accuracy (the accuracy of correctly classifying a fly into 1 of the 5 genotypes at each age group or 5 age groups for each genotype), and a pairwise classification accuracy (the accuracy of correctly classifying a fly into one of two correct genotypes at each age group). For detailed description of the methods we used to apply LDA to multivariate *Drosophila* data, please see West et al. (2015a).

Here, we hypothesize that *Drosophila* will be classified into their correct genotypic class at above-chance levels based on temporal contrast profiles, in line with previous findings using spatiotemporal profiles (West et al. 2015a).

### Overall Model Discrimination Accuracy

We first ran our full data set of 25 classes through the LDA to assess how well it could classify *Drosophila* when considering both their genotype and age. In this case, baseline (chance) performance was 4% (1/25). Next, to assess how well we could discriminate between *Drosophila* genotypes within each age group, our data were partitioned into five genotypes and LDA was applied with a 20% chance baseline (1/5). Finally, to assess how well we could classify between *Drosophila* at different ages within each genotype, our data were divided into five age groups within each genotype and analyzed using LDA, again with a 20% chance baseline (1/5).

The full overall classification accuracies for both LOO analysis and Monte Carlo resampling analysis for all three sets of data are presented in Table 1. The overall accuracy of our model in classifying *Drosophila* into their correct genotypic class differed depending on the age of the genotypes included in the model. The highest classifications occurred at 1 and 28 days of age. Although there was a slight decrease in accuracies when classifying *Drosophila* into their correct age within a genotype, the algorithm still performed above 20% chance baseline for all genotypes.

**Table 1.** Classification accuracies for LOO analysis and Monte Carlo resampling analysis

Class	LOO Classification	Monte Carlo Resampling
All 25 classes	24.8%	29.6%
1 day posteclosion	58%	68%
7 days posteclosion	52%	64%
14 days posteclosion	46%	54%
21 days posteclosion	48%	50%
28 days posteclosion	64%	70%
*w̄*	54%	54%
*DJ-1α*^Δ^*^72^*	38%	38%
*DJ-1β*^Δ^*^93^*	52%	52%
*PINK1^5^*	34%	50%
*dLRRK^ex1^*	26%	34%

Classification accuracy differs when flies are grouped by age and classified into genotype and when they are grouped by genotype and classified into age. Generally, both leave-one-out (LOO) and Monte Carlo resampling methods provide similar classification accuracies. *N* = 50 for per class (chance baseline 20%), except “All 25 classes” *N* = 250 (chance baseline 4%).

### N-Way Classification Accuracy

**Fig. 9.**
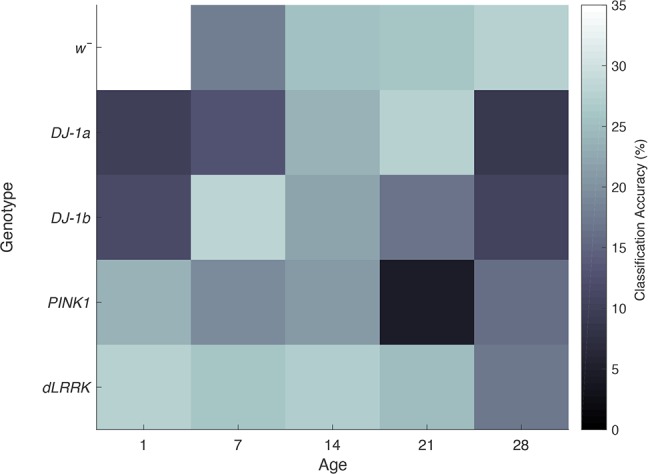
Linear discriminant analysis (LDA) can accurately discriminate between all 25 classes when they are included in the model. All classifications sit above 4% chance baseline, except for *PINK1^5^* at 21 days of age.

The confusion matrix was used to establish the accuracy of our LDA model to classify *Drosophila* into their correct genotypic class. Again, we investigated the precision of our model when all 25 classes were included in the model, with a 4% chance baseline (1/25). All classifications were reported above chance, bar *PINK1^5^* at 21 days of age. The highest accuracy was for *w̄ at* 1 day of age, where the model performed with 34.49% accuracy, whereas most other conditions were classified with ~25% accuracy. A profile of classification accuracies when all 25 classes are considered is presented in Fig. 9.

**Fig. 10.**
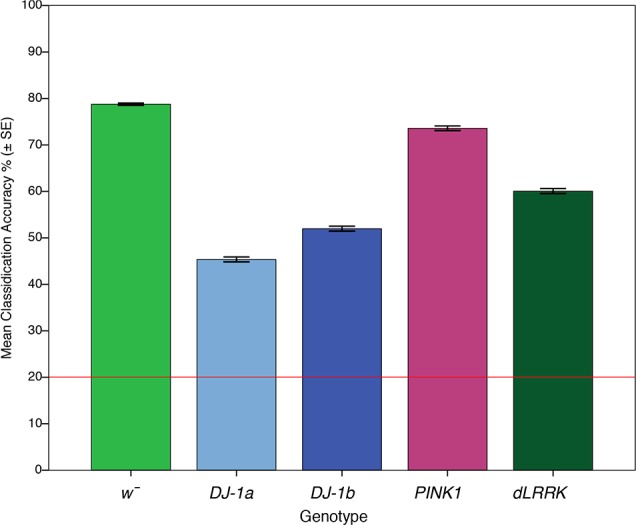
Classification of young flies by genotypic class using data from temporal contrast response profiles. Mean classification accuracies for N-way LDA of 5 genotypes at 1 day of age (*n* = 10 per genotype). The chance baseline is set at 20%, with mean classification accuracies between 45.5 and 78.8%.

Next, we assessed the ability of the classifier to accurately genotype *Drosophila* within each age group; thus five genotypes at each age were included in the model, with a 20% chance baseline (1/5). Our classification accuracy is deduced by normalizing our confusion matrix by dividing by the number of flies in each condition (*n* = 10). As illustrated in Fig. 10, at 1 day of age our model could classify *w̄* control flies into their correct genotypic class with 78.8% accuracy, whereas we could classify *DJ-1α*^Δ^*^72^* at 45.5% accuracy, *DJ-1β*^Δ^*^93^* at 52.9% accuracy, *PINK1^5^* at 73.6% accuracy and *dLRRK^ex1^* at 60.0% accuracy.

These accuracies shifted at 7 days of age, with our model classifying *w̄* with 29.8% accuracy, *DJ-1α*^Δ^*^72^* with 50.0% accuracy, *DJ-1β*^Δ^*^93^* with 64.7% accuracy, *PINK1^5^* with 62.2% accuracy, and *dLRRK^ex1^* at 46.9% accuracy. At 14 days of age our model could accurately classify *w̄* at 50.0% accuracy, *DJ-1α*^Δ^*^72^* at 68.1% accuracy, *DJ-1β*^Δ^*^93^* at 50.3% accuracy, *PINK1^5^* at 36.4% accuracy, and *dLRRK^ex1^* at 29.1% accuracy. At 21 days of age our model classified *w̄* at 58.35% accuracy, *DJ-1α*^Δ^*^72^* at 50.5% accuracy, *DJ-1β*^Δ^*^93^* at 50.2% accuracy, *PINK1^5^* at 25.7% accuracy, and *dLRRK^ex1^* 53.8% accuracy. At 28 days of age our model classified *w̄* with 53.7% accuracy, *DJ-1α*^Δ^*^72^* with 71.5% accuracy, *DJ-1β*^Δ^*^93^* with 62.6% accuracy, *PINK1^5^* with 55.1% accuracy, and *dLRRK^ex1^* at 46.35% accuracy.

### N-Way Classification Accuracy: Age

Here, our LDA model was used to classify *Drosophila* mutants into their correct age within a single genotype, with a 20% chance baseline (1/5). Comparatively, the model was generally weaker in accurately classifying into age compared with classifying into genotype, although all classifications exceeded chance baseline. Age N-Way classification accuracies for each genotype are presented in Table 2.

**Table 2.** Age N-Way classification accuracies for each genotype

	N-Way Classification Accuracy				
Genotype	1 day	7 days	14 days	21 days	28 days
*w̄*	81.3%	29.5%	32%	53.5%	53.5%
*DJ-1α*^Δ^*^72^*	26.6%	34.1%	50.0%	29.7%	48.4%
*DJ-1β*^Δ^*^93^*	55.3%	59.5%	51.0%	45.0%	57.3%
*PINK1^5^*	39.7%	49.1%	35.0%	27.2%	49.3%
*dLRRK^ex1^*	37.6%	23.7%	22.7%	30.2%	43.7%

Chance baseline: 20% (1/5). N-Way classification of flies into their correct age differs between genotypes. All classes can be classified above 20% chance baseline, with the highest accuracy sitting at 81.3% for 1-day-old *w̄* classifications (*n* = 10).

### Pairwise Classification Accuracy

To assess the accuracy of our model in classifying *Drosophila* between pairs of genotypes within each age group we bootstrapped our data through 1,000 iterations of a two-way classification analysis. Here, we assess the accuracy of the algorithm estimation in classifying a fly from a pair of genotypes into its correct class. Classification is significantly above chance when fewer than 5% of the bootstrapped two-way classification probabilities are 0.5 or greater.

As presented in Table 3, the algorithm classified 1-day-old *Drosophila* genotypes with accuracy between 73.7 and 94.1% (*P* < 0.05). Notably, all PD mutants could be accurately distinguished from *w̄* control flies.

**Table 3.** One-day-old Drosophila genotypes

	*w̄*	*DJ-1β*^Δ^*^93^*	*DJ-1α*^Δ^*^72^*	*dLRRK^ex1^*
*PINK1^5^*	94.1%*	84.7%*	78.8%*	88.9%*
*w̄*		86.3%*	75.8%*	77.6%*
*DJ-1β*^Δ^*^93^*			57.9%	73.7%*
*DJ-1α*^Δ^*^72^*				65.3%

Linear discriminant analysis (LDA) can accurately compute pairwise classifications between PD and control genotypes at 1 day of age (*n* = 10).

* *P <* 0.05.

As presented in Table 4, at 7 days of age the model had a reduction in the amount of significant comparisons, performing between 74.5 and 85.6% accuracy. At this age, the LDA could not accurately discriminate between any of the PD mutants and control flies.

**Table 4.** Comparisons at 7 days of age

	*w̄*	*DJ-1β*^Δ^*^93^*	*DJ-1α*^Δ^*^72^*	*dLRRK^ex1^*
*PINK1^5^*	69.9%	74.7%*	76.1%*	85.6%*
*w̄*		60.8%	60.5%	63.3%
*DJ-1β*^Δ^*^93^*			67.7%	76.3%*
*DJ-1α*^Δ^*^72^*				66.9%

LDA had a reduction in total significant comparisons at 7 days of age, and cannot accurately discriminate between any of the PD mutants compared against control flies (*n* = 10).

* *P <* 0.05.

At 14 days of age there appeared to be an overall improvement in pairwise classifications with significant pairwise classifications between 78.0 and 81.3% accuracy, as illustrated in Table 5.

**Table 5.** Comparisons at 14 days of age

	*w̄*	*DJ-1β*^Δ^*^93^*	*DJ-1α*^Δ^*^72^*	*dLRRK^ex1^*
*PINK1^5^*	61.7%	57.8%	78.6%*	79.2%*
*w̄*		78.4%*	78.0%*	79.9%*
*DJ-1β*^Δ^*^93^*			89.6%*	91.3%*
*DJ-1α*^Δ^*^72^*				52.1%

LDA can accurately compute pairwise classifications between PD and control genotypes at 14 days of age (*n* = 10). There are differences in accuracy compared with 7- and 1-day-old classifications.

* *P <* 0.05.

This held at 21 days of age, where our pairwise classification accuracy reached between 75.2 and 85.1% for significant comparisons, as illustrated in Table 6; however, there was a reduction in significant comparisons at this age.

**Table 6.** Comparisons at 21 days of age

	*w̄*	*DJ-1β*^Δ^*^93^*	*DJ-1α*^Δ^*^72^*	*dLRRK^ex1^*
*PINK1^5^*	63.3%	65.2%	75.2%*	52.9%
*w̄*		78.4%*	77.4%*	69.4%
*DJ-1β*^Δ^*^93^*			85.1%*	77.7%*
*DJ-1α*^Δ^*^72^*				60.6%

LDA can accurately compute pairwise classifications between PD and control genotypes at 21 day of age (*n* = 10); however, there are less significant comparisons compared with earlier ages.

* *P <* 0.05.

In line with our peak in overall model accuracy, our model was most accurate in classifying between flies at 28 days of age, with all possible comparisons statistically significant and sitting between 72.7 and 86.2% accuracy (Table 7). Similar to 1-day-old comparisons, all PD mutants could be accurately distinguished from *w̄* control flies at 28 days of age. We note that these statistics differ from the comparisons on the PCA simple effects analysis data, as will be addressed in our discussion.

**Table 7.** Comparisons at 28 days of age

	*w̄*	*DJ-1β*^Δ^*^93^*	*DJ-1α*^Δ^*^72^*	*dLRRK^ex1^*
*PINK1^5^*	78.9%*	78.7%*	79.7%*	73.7%*
*w̄*		86.2%*	81.0%*	75.6%*
*DJ-1β*^Δ^*^93^*			88.4%*	83.6%*
*DJ-1α*^Δ^*^72^*				72.7%*

LDA accurately computes pairwise classifications between all genotypes at 28 days of age (*n* = 10). All comparisons are significant and above 72.7% accuracy.

* *P <* 0.05.

## DISCUSSION

### Abnormal Gain Control in Early-Onset PD Drosophila models

We have demonstrated that abnormal gain control occurs in young EOPD mutants: *DJ-1α*^Δ^*^72^*, *DJ-1β*^Δ^*^93^*, and *PINK1^5^*. *Drosophila* with these mutations have significantly higher SSVEP response amplitudes compared with *w̄* controls at *day 1*. Notably, there appears to be no difference between response amplitudes of 1-day-old *w̄* controls and knockout of the fly *LRRK2* homologue *dLRRK^ex1^*. These results are consistent with previous studies and point to a common phenotype of abnormal gain control occurring in the current studied EOPD mutants and the *LRRK2-G2019S* late-onset mutant (Afsari et al. 2014; West et al. 2015b).

What common biological mechanism might explain these findings? Dopaminergic terminals are found in the *Drosophila* ommatidium, lamina, and medulla, where dopamine is thought to regulate contrast sensitivity, light adaptation, and circadian rhythms (Afsari et al. 2014; Chyb et al. 1999; Hirsh et al. 2010; Jackson et al. 2012; Nässel and Elekes 1992). Thus dopamine acts as a neuromodulator within the *Drosophila* visual system, effectively regulating neural response to visual excitation. PD-model flies may have less dopamine content, and/or fewer dopaminergic neurons, or disrupted dopamine signaling, though the reduction may depend on the environmental conditions (Navarro et al. 2014; Ng et al. 2012; Park et al. 2006; Wang et al. 2006). Any reduction in dopamine release will cause photoreceptors to respond faster and with greater amplitude (Chyb et al. 1999). This hyperactivity causes increased SSVEP amplitudes, manifesting as abnormal gain control. Humans, like flies, have retinal dopamine within the amacrine cells and inner border of the nuclear layer, where it is thought to be responsible for light adaptation, contour perception, and contrast sensitivity (Crooks and Kolb 1992; Dowling 1979; Witkovsky 2004). Human patients also show a reduction in retinal dopamine and report a range of low-level visual deficits, including poor contrast sensitivity and reduced light sensitivity (Archibald et al. Burn 2011; Beitz 2014; Chaudhuri and Schapira 2009; Weil et al. 2016). These homologies in retinal structure, function, and disease pathology point to the possibility that prodromal gain control abnormalities occur in human PD patients.

The response profile of wild-type *w̄ Drosophila* changes as a function of age. This genotype initially presented with comparatively low response amplitudes compared with EOPD mutants. The *w̄* response then increased between 1 and 7 days of age. This reflects the anatomical plasticity of the young *Drosophila* visual system. Young *w̄* flies are born with reduced visual sensitivity which then adapts to functional requirements, with visual maturity occurring between 4 and 7 days of age (Kral and Meinertzhagen 1989). It is important to note that all *Drosophila* included in our study are white eyed and thus share the *w̄* mutation. The increased sensitivity to visual stimuli we observe in EOPD mutants, and mutants’ unique developmental profiles, is due solely to the PD mutation.

### Excitotoxicity as a Pathological Phenotype in Parkinson’s Disease

Initially we saw no evidence of excitotoxic damage in the visual system of older PD flies. However, *Drosophila* in the laboratory experience a relatively stable visual environment: light levels are many orders of magnitude lower than those in the outside world and they are modulated according to a strict 12:12-h LD cycle. We theorized that purposeful visual stimulation of the PD *Drosophila* visual system may be necessary to induce excitotoxicity in the laboratory. To increase neural demand for energy we exposed flies to a rich visual environment that contained irregular bursts of high-intensity luminance modulations. This environment requires the photoreceptors to change both their firing rates and their mean sensitivity over relatively short time periods. Our hypothesis was that the abnormal gain control we observed in young EOPD flies would interact with an increase in neural activity to cause an excitotoxic cascade. Our data are consistent with this hypothesis: PD, but not *w̄* flies, showed reduced visual functionality after prolonged exposure to these visually demanding environments.

Our results provide evidence for an excitotoxic cascade in PD *Drosophila* mutants, with *DJ-1α*^Δ^*^72^*, *DJ-1β*^Δ^*^93^*, and *PINK1^5^* all showing a significant decrease in SSVEP amplitudes after 7 days of visual stimulation, with a minimum of 50% reduction in response. Surprisingly, the response amplitudes of *dLRRK^ex1^* mutants were also reduced, even though we did not observe abnormal gain control in this strain at 1 day of age.

We draw upon the previously established theory of excitotoxicity in PD explain the biological processes underlying our observed visual loss. Here, abnormal gain control interacts with a visually induced increase in neural demand. This causes an increase in ionic flux across the cell membrane which in turn results in extra demand for ATP from the ion exchange pumps. When mitochondria cannot meet this increased demand for ATP, they release reactive oxygen species (e.g., superoxide, hydrogen peroxide), so generating oxidative stress, which leads to autophagy, apoptosis and other forms of cell damage. This is then followed visual decline and eventual cell death (Hindle et al. 2013).

Mitochondrial dysfunction and oxidative stress appear to play a central role in PD pathogenesis (Bogaerts et al. 2008; Büeler 2009; Henchcliffe and Beal 2008; Schapira 2008). The present study has investigated *Drosophila* PD mutations in genes whose human homologues are associated with EOPD. In both humans and flies, *DJ-1* encodes a small protein that is thought to protect against oxidative stress and assist in mitochondrial regulation by acting as a sensor for reactive oxidative species (ROS) (Oswald et al. 2016). Subsequently, loss-of-function mutations in *DJ-1* appear to increase cell death in response to oxidative stress. Furthermore, animal studies have observed perturbations in dopamine release in *DJ-1* deficient animal models, although there is no physiological loss of dopamine neurons (Goldberg et al. 2005; Martella et al. 2011; Menzies et al. 2005; Meulener et al. 2005; Pisani et al. 2006; Yang et al. 2007). *PINK1* is a protein kinase with a mitochondrial targeting sequence and acts to maintain mitochondrial homeostasis in dopaminergic neurons (Park et al. 2006). Likewise, studies in *PINK1* animal models have found evidence for abnormal mitochondrial morphology and impaired dopamine release (Clark et al. 2006; Kitada et al. 2007; Park et al. 2006). Thus the protein products of both *DJ-1* and *PINK1* both play roles in the regulation of cellular energy production. However, loss-of-function mutations on these genes negatively impact mitochondria in different ways. Our data provide additional support for the hypothesis that mitochondrial impairment plays a role in the pathogenesis of genetic PD.

### Classification of Drosophila PD Genotype

Previously, we demonstrated that discriminant analysis is a useful tool that can accurately classify PD *Drosophila* into their correct genotypic class at 1 day of age (West et al. 2015a). Here, we build on this observation, establishing that variability within temporal contrast response profiles obtained from *Drosophila* can be used in a LDA to accurately classify *Drosophila* into their correct genotypic class at various ages with above chance accuracy. When all 25 classes were included in our model, our LOO classification accuracy sat at 24.8%, whereas our bootstrapped classification accuracy was 29.6% (chance baseline of 4%). Our LDA model also performed well when classifying five genotypes within a single age group. Highest classifications occurred at 1 day (Monte Carlo sampling accuracy of 68% and LLO accuracy of 58%) and 28 days of age (Monte Carlo sampling accuracy of 70% and LOO accuracy of 64%) with a baseline of 20%. This indicates that there are substantial differences between *Drosophila* genotypes at both 1 and 28 days of age.

When all 25 classes were included in our model, all classifications (except *PINK1^5^*) perform above a 4% chance baseline, with most classifications occurring with ~25% accuracy. There is substantial variation between PD *Drosophila* visual response throughout their lifespan, indicating that EOPD mutations have unique effects on *Drosophila* visual pathways at not only 1 day of age, but throughout the *Drosophila* lifespan. After our data were partitioned into five genotypes for each age group, we could classify *Drosophila* into their correct genotypic class with 29.8–78.8% accuracy over all possible age groups, with no classifications falling under the statistical chance baseline of 20%. Our results illustrate that mutants can be accurately classified into their correct genotypic class beyond 1 day of age, indicating there are subtle differences in how EOPD mutations affect *Drosophila* neural gain control, as will be discussed.

Although the N-Way classification accuracy decreased when the algorithm was required to classify *Drosophila* into their correct age within a single genotype, our model still performed above chance baseline. This is surprising considering the results of our first experiment, where, for the most part, within-genotype responses did not significantly differ over time. Our analysis was run on a reduced number of genotypes and flies [*n* = 10 and 5 genotypes, rather than *n* = 20 and 10 genotypes as per West et al. (2015a)], yet our model produced a consistently high classification accuracy, even with all 25 classes were included in the model. In West et al. (2015a), we varied temporal and spatial frequency but kept contrast fixed. We observed relatively little dependence on spatial frequency up to a hard cutoff that was associated with spatial sampling limits. Our use of contrast rather than spatial frequency in the experiments described here allows us to measure the full contrast sensitivity profile of each genotype and age, increasing the sensitivity of this multivariate visual biomarker for EOPD genes in *Drosophila*. Furthermore, our assay, when combined with LDA, is sensitive enough to detect small differences in the effect of EOPD mutations on *Drosophila* neural gain control. Our initial analysis found a substantial difference between *w̄* and EOPD mutants at 1 day of age; however, our LDA results indicate that these mutations have their own subtle effects on neural gain control across *Drosophila* lifespan. Our findings carry an important implication. As noted, *DJ-1* acts as a ROS sensor, whereas *PINK1* acts to maintain mitochondrial homeostasis in dopaminergic neurons (Lavara-Culebras et al. 2010; Oswald et al. 2016; Park et al. 2006). The ability of our LDA to accurately distinguish between mutations on these genes indicates each mutation uniquely impacts the underlying cellular processes, thereby causing a subtle, dissimilar neural responses across *Drosophila* lifespan that then results in a common pathogenic outcome of visual loss and cell death.

A key benefit of using *Drosophila* as disease model is their convenience for early-stage drug testing due to their fecundity and fast generation time. It is advantageous to have phenotypic expression of PD mutations at early stages of *Drosophila* lifespan as this supports their utility as an initial model for the rapid testing of neuroactive drugs that have the potential to treat human disease. Like *Drosophila*, perturbations in contrast sensitivity occur in human PD patients due to reduced dopamine levels within the retina (Harnois and Di Paolo 1990). Our current findings may correspond to the changes seen in human PD patients, although there is obvious difficulty in assessing whether a prodromal abnormal gain control occurs in the early stages of pregenotyped PD patients. We believe that it may be possible for LDA to classify human PD patients’ genotype based on multivariate SSVEP response profiles as measured by electroencephalogram. This would have the potential to assist in early PD diagnosis, genotypic classification, and disease expression. Our next step is to investigate *Drosophila* response to additional low-level visual parameters such as chromatic contrast and orientation in order to deduce whether a similar biomarker can be established in human PD patients.

Together, our experiments have uncovered abnormal gain control and an excitotoxic cascade as a common pathological phenotype in three EOPD mutations: *DJ-1α*^Δ^*^72^*, *DJ-1β*^Δ^*^93^*, and *PINK1^5^*. In addition to furthering the link between abnormal gain control and excitotoxicity in genetic forms of PD, our findings have built on the utility of LDA in genotyping *Drosophila* based on multivariate response profiles. Furthermore, we have illustrated that there are variations in how these EOPD mutations affect neural gain control across *Drosophila* lifespan, indicating that these mutations have unique effects upon underlying cellular processes that lead to a common outcome: visual loss and cell death. Overall, it appears that these PD-related mutations are heterochronic: mutations lead to stronger neural signaling (increased sensory response may be beneficial in escaping behavior) in young flies but are detrimental in older flies (a loss of vision would hinder escape behavior) (Himmelberg et al. 2018). Should these findings in fly models prove applicable to the human situation, it would suggest that prodromal PD may be linked to changes in central nervous system processing that could, potentially, confer advantages in early life at the cost of degenerative disease in old age.

## GRANTS

M. M. Himmelberg was supported by the European Union’s Horizon 2020 research and innovation program under the Marie Sklodowska-Curie grant agreement no. 641805. R. J. H. West developed the original equipment and methods under support from the Wellcome Trust and the York Centre for Chronic Diseases and Disorders (C2D2) (ref. 097829/Z/11/A).

## DISCLOSURES

No conflicts of interest, financial or otherwise, are declared by the authors.

## AUTHOR CONTRIBUTIONS

M.M.H., R.J.W., C.J.E., and A.R.W. conceived and designed research; M.M.H. performed experiments; M.M.H., R.J.W., C.J.E., and A.R.W. analyzed data; M.M.H., R.J.W., C.J.E., and A.R.W. interpreted results of experiments; M.M.H., R.J.W., C.J.E., and A.R.W. prepared figures; M.M.H. drafted manuscript; M.M.H., R.J.W., C.J.E., and A.R.W. edited and revised manuscript; M.M.H., R.J.W., C.J.E., and A.R.W. approved final version of manuscript.

## APPENDIX

Table A1 shows *P* values for simple effects comparing between genotypes within each age group, and Table A2 shows *P* values for simple effects comparing between age groups within each genotype.

**Table A1.** Simple effects P values comparing between genotype at each age

Age	*w̄*	*DJ-1β*^Δ^*^93^*	*DJ-1α*^Δ^*^72^*	*dLRRK^ex1^*
1 day				
* PINK1^5^*	*P* < 0. 001*	*P* = 0.724	*P* = 0.048*	*P* < 0.001*
* w̄*		*P* < 0.001*	*P* = 0.006*	*P* = 0.302
* DJ-1β*^Δ^*^93^*			*P* = 0.892	*P* = 0.083
* DJ-1α*^Δ^*^72^*				*P* = 0.852
7 days				
* PINK1^5^*	*P* = 0.208	*P* = 0.158	*P* = 0.185	*P* = 0.004*
* w̄*		*P* = 0.208	*P* = 1.000	*P* = 1.000
* DJ-1β*^Δ^*^93^*			*P* = 1.000	*P* = 0.940
* DJ-1α*^Δ^*^72^*				*P* = 0.917
14 days				
* PINK1^5^*	*P* = 1.000	*P* = 0.221	*P* = 0.042*	*P* = 0.019*
* w̄*		*P* = 0.064	*P* = 0.156	*P* = 0.080
* DJ-1β*^Δ^*^93^*			*P* < 0. 001*	*P* < 0.001*
* DJ-1α*^Δ^*^72^*				*P* = 1.000
21 days				
* PINK1^5^*	*P* = 0.897	*P* = 0.737	*P* = 0.440	*P* = 0.862
* w̄*		*P* = 0.052	*P* = 0.999	*P* = 1.0
* DJ-1β*^Δ^*^93^*			*P* = 0.006*	*P* = 0.042*
* DJ-1α*^Δ^*^72^*				*P* = 1.000
28 days				
* PINK1^5^*	*P* = 0.515	*P* = 1.000	*P* = 0.010*	*P* = 0.275
* w̄*		*P* = 0.440	*P* = 0.753	*P* = 1.000
* DJ-1β*^Δ^*^93^*			*P* = 0.007*	*P* = 0.222
* DJ-1α*^Δ^*^72^*				*P* = 0.937

* *P <* 0.05.

**Table A2.** Simple effects P values comparing between age within each genotype

Genotype	7 days	14 days	21 days	28 days
*w̄*				
1 day	*P* = 0.001*	*P* < 0.001*	*P* = 0.05*	*P* < 0.001*
7 days		*P* = 0.811	*P* = 1.000	*P* = 1.000
14 days			*P* = 0.372	*P* = 0.991
21 days				*P* = 0.951
*DJ-1α^Δ72^*				
1 day	*P* = 1.000	*P* = 1.000	*P* = 1.000	*P* = 1.000
7 days		*P* = 0.988	*P* = 0.938	*P* = 0.988
14 days			*P* = 1.000	*P* = 1.000
21 days				*P* = 1.000
*DJ-1β^Δ93^*				
1 day	*P* = 0.988	*P* = 0.005*	*P* = 0.691	*P* = 0.507
7 days		*P* < 0.001*	*P* = 0.178	*P* = 0.099
14 days			*P* = 0.427	*P* = 0.609
21 days				*P* = 1.000
*PINK1^5^*				
1 day	*P* = 1.000	*P* = 1.000	*P* = 0.768	*P* = 1.000
7 days		*P* = 1.000	*P* = 0.698	*P* = 1.000
14 days			*P* = 0.923	*P* = 1.000
21 days				*P* = 0.634
*dLRRK^ex1^*				
1 day	*P* = 0.998	*P* = 0.997	*P* = 0.852	*P* = 0.242
7 days		*P* = 1.000	*P* = 0.999	*P* = 0.733
14 days			*P* = 0.1.000	*P* = 0.806
21 days				*P* = 0.993

* *P <* 0.05.
